# Patient education on subacromial impingement syndrome: Reliability and educational quality of content available on Google and YouTube

**DOI:** 10.1007/s00132-022-04294-x

**Published:** 2022-08-22

**Authors:** Malik Jessen, Christina Lorenz, Elisabeth Boehm, Stefan Hertling, Maximilian Hinz, Jan-Philipp Imiolczyk, Carsten Pelz, Yacine Ameziane, Sebastian Lappen

**Affiliations:** 1 Department of Trauma Surgery, Klinikum rechts der Isar, Technical University of Munich, Munich, Germany; 2 Department of Trauma Surgery, University Hospital Zurich, Zurich, Switzerland; 3 Department of Orthopaedics and Trauma Surgery, Musculoskeletal University Center Munich (MUM), University Hospital, LMU Munich, Munich, Germany; 4 Orthopedic Department of the Waldkliniken Eisenberg, University Hospital Jena, Eisenberg, Germany; 5 Department of Orthopaedic Sports Medicine, Klinikum rechts der Isar, Technical University Munich, Ismaninger Str. 22, 81675 Munich, Germany; 6 Center for Musculoskeletal Surgery, Charité University Medicine, Berlin, Germany; 7 Department of Hematology, Oncology and Tumor Immunology, Charité University Medicine, Berlin, Germany; 8 Orthopedic Practice Clinic (OPPK), Münster, Germany

**Keywords:** Video analysis, Qualitative research, Shoulder pain, Health literacy, Internet, Videoanalyse, Informationsgehalt, Schulterschmerzen, Gesundheitskompetenz, Internet

## Abstract

**Objective:**

The purpose of this study was to assess the reliability and educational quality of content available on Google and YouTube regarding subacromial impingement syndrome (SAIS).

**Methods:**

Google and YouTube were queried for English and German results on SAIS using the search terms “shoulder impingement” and the German equivalent “Schulter Impingement”. The analysis was restricted to the first 30 results of each query performed. Number of views and likes as well as upload source and length of content were recorded. Each result was evaluated by two independent reviewers using the *Journal of the American Medical Association* (JAMA) benchmark criteria (score range, 0–5) to assess reliability and the DISCERN score (score range, 16–80) and a SAIS-specific score (SAISS, score range, 0–100) to evaluate educational content.

**Results:**

The 58 websites found on Google and 48 videos found on YouTube were included in the analysis. The average number of views per video was 220,180 ± 415,966. The average text length was 1375 ± 997 words and the average video duration 456 ± 318 s. The upload sources were mostly non-physician based (74.1% of Google results and 79.2% of YouTube videos). Overall, there were poor results in reliability and educational quality, with sources from doctors having a significantly higher mean reliability measured in the JAMA score (*p* < 0.001) and educational quality in DISCERN (*p* < 0.001) and SAISS (*p* = 0.021). There was no significant difference between German and English results but texts performed significantly better than videos in terms of reliability (*p* = 0.002) and educational quality (*p* < 0.001).

**Conclusion:**

Information on SAIS found on Google and YouTube is of low reliability and quality. Therefore, orthopedic health practitioners and healthcare providers should inform patients that this source of information may be unreliable and make efforts to provide patients with higher quality alternatives.

Level of evidence: IV, case series.

**Electronic supplementary material:**

The online version of this article (10.1007/s00132-022-04294-x) contains supplementary material, which is available to authorized users.

## Abstract (de)

**Zielsetzung:**

Ziel dieser Studie war es, die Reliabilität und Informationsqualität der auf Google und YouTube verfügbaren Inhalte zum subakromialen Impingement-Syndrom (SAIS) zu bewerten.

**Methoden:**

Die Webseiten Google und YouTube wurden mit den Begriffen „shoulder impingement“ und „Schulter-Impingement“ durchsucht. Eingeschlossen wurden jeweils die ersten 30 Suchergebnisse. Erfasst wurden die Anzahl der Aufrufe und „Likes“ sowie die Upload-Quelle und die Länge des Inhalts. Jedes Suchergebnis wurde von zwei unabhängigen Untersuchern anhand der Benchmark-Kriterien des *Journal of the American Medical Association* (JAMA; Wertebereich 0–5) bewertet, um die Reliabilität zu beurteilen, sowie anhand des DISCERN-Scores (16–80 Punkte) und eines SAIS-spezifischen Scores (SAISS, 0–100 Punkte), um den Informationsgehalt in Bezug auf das SAIS zu ermitteln.

**Ergebnisse:**

Es konnten 58 Textinhalte von Google sowie 48 Videos von YouTube in der Auswertung berücksichtigt werden. Die durchschnittliche Anzahl der Aufrufe pro Video betrug 220.180 ± 415.966. Die durchschnittliche Textlänge betrug 1375 ± 997 Wörter und die durchschnittliche Videodauer 456 ± 318 s. Die meisten Quellen wurden von nichtärztlichen Autoren verfasst (74,1 % der Google-Ergebnisse und 79,2 % der YouTube-Videos). Die Reliabilität und der Informationsgehalt der Quellen wurden insgesamt als schlecht bewertet, wobei die Quellen von ärztlichen Autoren eine signifikant höhere Reliabilität, gemessen am JAMA-Score (*p* < 0,001), und eine höhere Informationsqualität gemäß DISCERN (*p* < 0,001) und SAISS (*p* = 0,021) aufwiesen. Es gab keinen signifikanten Unterschied in Bezug auf die Sprache, jedoch bezüglich des Mediums. Textinhalte schnitten sowohl in der Reliabilität (*p* = 0,002) als auch in deren Informationsgehalt (*p* < 0,001) signifikant besser ab als Videos.

**Schlussfolgerung:**

Die auf Google und YouTube analysierten Quellen über SAIS sind von geringer Reliabilität und geringem Informationsgehalt. Daher sollten Ärzte/Behandler ihre Patienten darüber informieren, dass selbstgefundene Informationen aus Google- und YouTube-Suchergebnissen möglicherweise unzuverlässig und unvollständig sind. Es sollten den Patienten neben dem persönlichen Aufklärungsgespräch alternative Informationsquellen angeboten werden.

Evidenzgrad: IV, Fallserie.

## Introduction

Patient health literacy has been proven to be one of the most important indicators of health status [1, 2]. The Internet is gaining an increasingly important role in the acquisition of health-related information as an easily accessible and frequently used source [3–5]. For a large proportion of patients, the Internet has even become the primary source of information on medical issues [6, 7]. With over 50% of the world’s population now having Internet access [8], it is likely that the use of the Internet to obtain medical information will continue to increase.

YouTube and Google are the two most visited websites worldwide [9] and are frequently used by patients looking for medical information [7, 10]. However, like content from other online resources, the content found on Google and YouTube lacks an editorial process, often resulting in poor quality content or inaccuracy [11–13]. As there is therefore a risk of inaccurate content and misinformation being disseminated, clinicians should be aware of these resources and their quality.

Subacromial impingement syndrome (SAIS) accounts for 44–65% of all shoulder complaints in primary care and is therefore considered one of the most common shoulder disorders [14]. The prevalence is estimated to be between 7 and 26% of the general population [15]. With the number of surgical interventions continuously growing, SAIS is of great importance to health systems worldwide [16].

While quality-based studies on online information regarding orthopedic topics, such as kyphosis [8], anterior cruciate ligament (ACL) ruptures [17], or meniscus lesions [18] have already been performed, the reliability and educational quality of the content found online on SAIS have not yet been evaluated. Therefore, the purpose of this study was to evaluate the reliability and educational quality of content found on Google and YouTube concerning SAIS and to identify factors predicting higher reliability and quality. We hypothesized that (1) most of the content would be of low reliability and educational quality, (2) text content found on Google and content published by physicians would be of higher quality than videos found on YouTube and content by non-physicians, and (3) language and popularity would not be indicators of high quality.

## Methods

### Search strategy

**Fig. 1:**
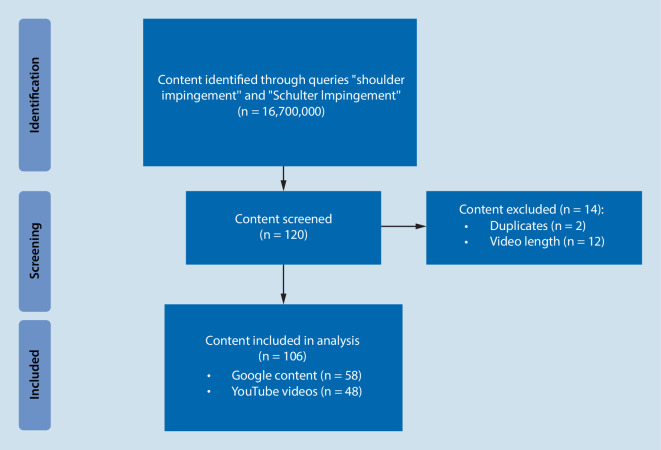
Flowchart for content selection

The YouTube online library (https://www.youtube.com) as well as the Google search engine website (https://www.google.com) were queried on 23 May 2021 using both English (“shoulder impingement”) and German (“Schulter Impingement”) search items. Beforehand, all settings of the browser used were set to default and no user account was logged in on either website. The standard search setting of “relevance” was used on both websites. The first 30 items of each search were analyzed, which was considered sufficient, as 90% of search engine users do not look beyond the first three pages of search results [19]. Only freely accessible content was eligible for inclusion. Content was excluded if it was of other language than English of German. Additionally, videos shorter than 2 min and text sources with less than 100 words were also excluded. The search methodology is shown in Fig. 1.

### Data review

Each item of content was analyzed independently by two reviewers. The following characteristics were documented for each content: (1) text length in number of words or video duration in minutes, (2) source of publication and (3) date of upload. The sources were categorized as follows: (1) physician, (2) physical therapist, (3) trainer, (4) other non-healthcare providers and (5) unknown authorship. Additionally, the number of views and the number of likes was extracted for YouTube videos.

### Evaluation of video accuracy and reliability

To assess content accuracy and reliability, the *Journal of the American Medical Association* (JAMA) benchmark criteria were used, which consist of four individual criteria (Table 1; [20]). Each item is rated with 0 (does not meet the desired criteria) or 1 point (meets the desired criteria), resulting in a total score between 0 and 4. Higher score numbers indicate greater accuracy and reliability of the content evaluated.

**Table 1:** JAMA benchmark criteria [20]

Criteria	Description
Authorship	Author and contributor credentials and their affiliations should be provided
Attribution	All copyright information should be clearly listed, and references and sources for content should be stated
Currency	The initial date of posted content and dates of subsequent updates to content should be provided
Disclosure	Conflicts of interest, funding, sponsorship, advertising, support, and video ownership should be fully disclosed

*JAMA* Journal of the American Medical Association

### Evaluation of educational quality

The educational quality was evaluated using the DISCERN scoring system (Quality Criteria for Consumer Health Information; Table 2) developed by an expert group at Oxford University [21]. The scale consists of three sections involving 16 questions, with each question being scored between 1 and 5 points. The first section (questions 1–8) assesses the reliability of the content, the second section (questions 9–15) focuses on the quality of information concerning treatment options and the third section (question 16) contains an overall evaluation of the content. The total score varies between 5 and 80 points, with higher scores indicating higher quality.

**Table 2:** DISCERN items [21]

*SECTION 1—Reliability*	
1	Is the publication reliable?
2	Does it achieve its aims?
3	Is it relevant?
4	Is it clear what sources of information were used to compile the publication (other than the author or producer)?
5	Is it clear when the information used or reported in the publication was produced?
6	Is it balanced and unbiased?
7	Does it provide details of additional sources of support and information?
8	Does it refer to areas of uncertainty?
*SECTION 2—Quality of information on treatment*	
9	Does it describe how each treatment works?
10	Does it describe the benefits of each treatment?
11	Does it describe the risks of each treatment?
12	Does it describe what would happen if no treatment is used?
13	Does it describe how the treatment choices affect overall quality of life?
14	Is it clear that there may be more than one possible treatment choice?
15	Does it provide support for shared decision-making?
*SECTION 3—Overall rating*	
16	Based on the answers to all of the above questions, rate the overall quality of the publication as a source of information about treatment choices

Since there is no evaluation tool for the quality assessment on information specifically for SAIS, the authors created a novel scoring system (referred to as the subacromial impingement syndrome score, SAISS) based on a literature review and expert opinion [22–27]. Comparable approaches were used to create scoring systems in previous studies [11, 28, 29]. The aim of developing the SAISS was to be able to evaluate content on SAIS in as much detail as possible. The number of points given per item varies from 1 to 5 points, depending on the relevance of the item as assessed by the authors. The SAISS consisted of the following six components: definition (5 points), etiology/pathogenesis (20 points), common patient presentations and symptoms (15 points), diagnosis (19 points), differential diagnosis (10 points) and treatment options (31 points) (Table 3). A maximum score of 100 points can be achieved, with a higher score indicating a better educational quality. The evaluation sheet for determining the score is included in *Supplement 1*.

**Table 3:** Subacromial impingement syndrome score (SAISS)

Criteria	Points
Definition	5
Etiology/pathogenesis	20
Symptoms	15
Diagnostics	19
Differential diagnosis	10
Therapy	31
Total	100

### Statistical analysis

The data were analyzed using IBM SPSS Statistics, version 28 (SPSS, Chicago, IL, USA). Descriptive statistics were used to quantify video characteristics as well as score results. Unpaired t‑test (for normally distributed data) and Mann-Whitney-U-tests (for non-normally distributed data) were used to determine whether video reliability and quality differed based on language, format, source, or popularity (number of views and likes). Multivariate linear regression analyses were performed to determine the influence of YouTube video popularity (number of views and likes) on reliability and quality. Interobserver agreement of JAMA, DISCERN, and SAISS was evaluated using the intraclass correlation coefficient (ICC) followed by the 95% confidence interval. *p* < 0.05 values were considered statistically significant. Pearson’s correlation analysis was used to examine the relationship between number of views/likes of YouTube videos and DISCERN, JAMA and SAISS scores. The criterion for statistical significance was *p* < 0.05 in all evaluations.

## Results

**Fig. 2:**
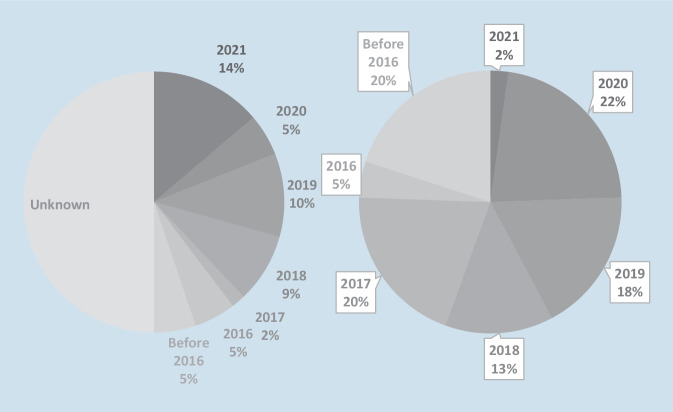
Publication date for Google (*left*) and YouTube (*right*) content by percentage distribution

Of the initial 120 search results generated by Google and YouTube searches, 2 Google sources and 12 YouTube videos did not meet the inclusion criteria and were excluded from the analysis (Fig. 1). Ultimately, 48 YouTube videos and 58 Google sources were included. The mean text length was 1375 ± 997.16 words and the average video duration was 456 ± 318 s. The majority of the content was not provided by physicians, accounting for 74.1% and 79.2% of uploaders for Google and YouTube videos, respectively. Unknown authors uploaded most of the content found on Google (60.3%) while physical therapists uploaded most of the YouTube videos (43.8%). Half of the content found on Google did not specify the upload date, while most of the YouTube videos were uploaded in 2020. Figure 2 provides an overview. The mean number of video views was 220,180 ± 415,966, and collectively, the 48 videos were viewed 10,568,639 times. The videos received a mean number of 3928 ± 11,551 likes (range 24–76,662).

The mean JAMA score for Google and YouTube content was 1.8 ± 1.3 and 2.5 ± 0.6, respectively. Google content showed a mean DISCERN of 48.5 ± 10.5 while YouTube showed a mean DISCERN of 33.2 ± 6.7. The mean SAISS for Google and YouTube content was 45.3 ± 16.2 and 18.5 ± 12.4, respectively. Highest mean scores were achieved in the subcategories “Definition” and “Symptoms”, whereas the lowest mean scores were given in “Differential diagnoses” and “Therapy”. Table 4 provides an overview of the scores obtained. Highest SAISS score for Google sources was 84.5 points [30] and 54.5 points for YouTube videos [31]. Intraobserver reliability was high with an ICC of 0.96 (95% confidence interval, 0.94–0.97) for JAMA, 0.98 (95% confidence interval, 0.97–0.99) for DISCERN, and 0.98 (95% confidence interval, 0.97–0.99) for SAISS.

**Table 4:** Mean quality and reliability scores

	Google	YouTube
*JAMA*	*1.8* *±* *1.3*	*2.5* *±* *0.6*
*DISCERN*	*48.5* *±* *10.5*	*33.1* *±* *6.7*
DISCERN a	26.8 ± 6.2	21.0 ± 4.3
DISCERN b	18.3 ± 5.1	10.1 ± 2.9
DISCERN c	3.4 ± 1.1	2.1 ± 0.7
*SAISS*	*45.3* *±* *16.2*	*18.6* *±* *12.4*
Definition	4.6 (91.0%) ± 1.2	3.3 (66.7%) ± 2.4
Etiology	10.2 (51.1%) ± 4.5	5.3 (26.5%) ± 4.8
Symptoms	9.5 (63.3%) ± 2.8	4.9 (32.7%) ± 3.2
Diagnostics	8.7 (45.9%) ± 5.5	1.8 (9.7%) ± 3.1
Differential diagnosis	1.0 (9.5%) ± 2.0	0.5 (4.5%) ± 1.0
Therapy	11.3 (36.6%) ± 6.6	2.7 (8.8%) ± 2.9

*NOTE*: Data are presented as mean (percentage of maximum points) ± standard deviation

*JAMA* Journal of the American Medical Association, *DISCERN* Quality Criteria for Consumer Health Information, *SAISS* Subacromial Impingement Syndrome Score

There was no significant difference between German and English results for JAMA (*p* = 0.922), DISCERN (*p* = 0.450) or SAISS (*p* = 0.572). However, videos scored significantly better for JAMA (*p* = 0.002), while texts scored higher for DISCERN (*p* < 0.001) and SAISS (*p* < 0.001). Content uploaded by physicians showed significantly higher JAMA (*p* < 0.001), DISCERN (*p* < 0.001) and SAISS scores (*p* = 0.021) compared to content uploaded by non-physicians.

Neither the number of views nor the number of likes were found to be independent predictors for JAMA, DISCERN or SAISS (Table 5*)*. Interestingly, the high correlation (Pearson’s r of 0.773) between the number of views and the number of likes was highly significant (*p* < 0.0001).

**Table 5:** Pearson correlations matrix for evaluating the relationship between number of views/likes of Youtube videos (*n* = 48) and DISCERN, JAMA, SAISS scores

Pearson correlations matrix					
		DISCERN	JAMA	SAISS	Number of likes
**Number of views**	Pearson’s r	−0.127	−0.073	0.202	0.773****
	*p*-value (two-tailed)	0.391	0.623	0.168	< 0.0001
**Number of likes**	Pearson’s r	−0.093	0.095	−0.011	–
	*p*-value (two-tailed)	0.531	0.521	0.943	–

*JAMA* Journal of the American Medical Association, *DISCERN* Quality Criteria for Consumer Health Information, *SAISS* Subacromial Impingement Syndrome Score

* *p* < 0.05, ** *p* < 0.01, *** *p* < 0.001, **** *p* < 0.0001

## Discussion

The principal findings of this study were that (1) content found on Google and YouTube on SAIS was of low to intermediate reliability and educational quality; (2) SAIS-related content was of great interest with a total of 10,568,639 views of only the 48 videos included; (3) physicians only created a small part of the content, but offered significantly better reliability and educational quality; (4) content on Google had a lower reliability but higher educational quality than YouTube videos; and (5) no conclusions could be drawn about the quality of the content based on the language or popularity of the content.

In addition to the established scoring systems JAMA and DISCERN, a self-developed SAISS was used in this study. While there is still no full agreement on the selection of scores for assessing health information available online, JAMA and DISCERN are currently among the most widely utilized tools due to their ease of use [17, 32, 33]. However, the authors considered it necessary to also use a score that captures information specific to SAIS. Many comparable studies used such specific scoring tools [17, 28, 34, 35]. Although these scoring systems are not validated, they allow a more precise analysis of the included content.

The results of our analysis were in agreement with comparable studies that reported poor reliability and quality on medical content available online [36–38]. The mean JAMA scores in the present study for Google and YouTube content were 1.8 ± 1.3 and 2.5 ± 0.6, respectively. Similar results were found in comparable studies on the anterior cruciate ligament (ACL) (2.4) [17], posterior cruciate ligament (PCL) (2.02) [29], meniscus (1.55) [18], kyphosis (1.34) [8], and disc herniation (1.7) [39]. The average DISCERN values in our analysis were also similar to those of comparable studies. The mean DISCERN of 48.5 ± 10.5 for Google content and 33.2 ± 6.7 for YouTube content determined in the present study were similar to that of a comparable study on disc herniation (30.8) [39], while studies on ACL and lower back pain using the modified brief DISCERN tool also showed low quality values [17, 32]. The mean SAISS for Google and YouTube content were 45.3 ± 16.2 and 18.5 ± 12.4 out of a maximum of 100 possible points, respectively. Low pathology-specific scores were also found in studies concerning ACL (5.5 of a maximum of 25 points) [17], PCL (2.9 of a maximum of 22 points) [29] and meniscus (3.67 of a maximum of 20 points) [18]. An in-depth look at the subcategories of SAISS showed that there was a particular lack of content that would enable an adequate presentation of the differential diagnoses of shoulder pain. This lack of information about causes other than SAIS is worrying as it can lead to patients overlooking other possible causes of their shoulder pain. Furthermore, only few points were achieved regarding treatment options. A study by MacLeod et al. [28] analyzed videos on femoral acetabular impingement and showed particular deficiencies regarding surgical complications and follow-up care, while similar deficiencies were found in our analysis. However, this is of particular importance for patients considering surgical treatment. Taken together, based on the results of the present and the previously discussed studies on other orthopedic pathologies, both Google and YouTube may not be sufficient resources to educate patients due to the poor reliability and quality of the content.

In total, the included YouTube videos were viewed 10,568,639 times and the average number of views per video in our study was 220,171 views. The topic of SAIS appears to reach a large online audience with mostly higher number of views than those of comparable studies, e.g. on injuries of the ACL (average 165,361 views per video) [17], PCL (average 50,477.9 views per video) [29], meniscus (a total of 14,141,285 views of 50 videos, average 288,597.7 views per video) [18], herniated discs (an average of 423,472 views per video) [39] and kyphosis (a total of 6,582,221 views of 50 videos) [8]. This underlines the importance of promoting accurate educational content for patients who use Google and YouTube as a source for healthcare information.

In the present study, content with an upload source categorized as physician showed significantly better reliability and educational quality. However, physicians only uploaded 25.7% of the content found on Google and 20.8% of the videos found on YouTube which correlates with results from comparable studies [18, 28, 29]. A video’s popularity measured in the number of views and the number of likes was no independent predictor for neither reliability nor educational quality (Table 5*)*. This underscores the difficulty for patients to find quality content as they cannot rely on the most popular sources. While this effect could only be measured for videos as Google does not display the number of views and does not have a rating system, it cannot be completely ruled out that the popularity of text sources would have shown different results. However, the authors consider it likely that this effect measured for videos can also be transferred to text sources.

### Limitations

One of the limitations of the present study is that the data were collected on a single day in a single geolocation and can therefore only be viewed as a snapshot of the information available at a given time. In addition, accounting for the large amount of content available online, only a fraction of this has been examined. However, most Internet users search no further than the first 3 result pages [40] and the aim of this study was to analyze the results that patients come across rather than analyze all possible information on the Internet. The low results from comparable studies also suggest that the consistently low values for SAIS content are representative of most SAIS content found online. There is also the possibility of some selection bias as Google and YouTube were the only websites queried. However, since Google and YouTube are the two most frequently used websites worldwide [7, 10], we consider their use to be suitable and clinically relevant as many patients access this content. Furthermore, by only including text sources of at least 100 words or videos with a minimum duration of 2 min, it is possible that content of different reliability and quality has been excluded. However, the authors chose these exclusion criteria to ensure that the content reviewed by this study was of reasonable length and therefore more likely to be a valid patient resource. Another limitation are the scores used as JAMA and DISCERN are not validated, and the SAISS is a score developed specifically for this study. However, JAMA and DISCERN are frequently used tools [8, 17, 18, 29] just as the process of developing pathology-specific scores has been done often in similar studies [17, 18, 29].

## Conclusion

The information found on Google and YouTube on SAIS is of poor reliability and quality. Given the role of the Internet as a source of medical content, healthcare professionals should be aware of the potential for misinformation and should be able to identify or, if necessary, provide alternative material of good quality.

## Caption Electronic Supplementary Material

Subacromial Impingement Syndrom Score Evaluation Sheet

## Declarations

For this article no studies with human participants or animals were performed by any of the authors. All studies mentioned were in accordance with the ethical standards indicated in each case.

## References

[1] Baker DW (1997). The relationship of patient reading ability to self-reported health and use of health services. Am J Public Health.

[2] Badarudeen S, Sabharwal S (2010). Assessing readability of patient education materials: current role in orthopaedics. Clin Orthop Relat Res.

[3] Tan SS, Goonawardene N (2017). Internet health information seeking and the patient-physician relationship: a systematic review. J Med Internet Res.

[4] Pollock W, Rea PM (2019). The use of social media in anatomical and health professional education: a systematic review. Adv Exp Med Biol.

[5] Khamis N (2018). Undergraduate medical students’ perspectives of skills, uses and preferences of information technology in medical education: a cross-sectional study in a Saudi Medical College. Med Teach.

[6] Baker JF (2010). Prevalence of Internet use amongst an elective spinal surgery outpatient population. Eur Spine J.

[7] Madathil KC (2015). Healthcare information on YouTube: a systematic review. Health Informatics J.

[8] Erdem MN, Karaca S (2018). Evaluating the accuracy and quality of the information in Kyphosis videos shared on YouTube. Spine.

[9] https://www.similarweb.com/top-websites/. Accessed 10 Dec 2021

[10] O’Carroll AM (2015). Information-seeking behaviors of medical students: a cross-sectional web-based survey. JMIR Med Educ.

[11] Wang D (2017). Evaluation of the quality, accuracy, and readability of Online patient resources for the management of articular cartilage defects. Cartilage.

[12] Badarudeen S, Sabharwal S (2008). Readability of patient education materials from the American Academy of Orthopaedic Surgeons and Pediatric Orthopaedic Society of North America web sites. J Bone Joint Surg Am.

[13] Shah AK, Yi PH, Stein A (2015). Readability of orthopaedic oncology-related patient education materials available on the internet. J Am Acad Orthop Surg.

[14] Bhattacharyya R, Edwards K, Wallace AW (2014). Does arthroscopic sub-acromial decompression really work for sub-acromial impingement syndrome: a cohort study. BMC Musculoskelet Disord.

[15] Luime JJ (2004). Prevalence and incidence of shoulder pain in the general population; a systematic review. Scand J Rheumatol.

[16] Vitale MA (2010). The rising incidence of acromioplasty. J Bone Joint Surg Am.

[17] Cassidy JT (2018). YouTube provides poor information regarding anterior cruciate ligament injury and reconstruction. Knee Surg Sports Traumatol Arthrosc.

[18] Kunze KN (2020). Quality of online video resources concerning patient education for the meniscus: a youtube-based quality-control study. Arthroscopy.

[19] (2006) iProspect Search Engine User Behavior Study. http://district4.extension.ifas.ufl.edu/Tech/TechPubs/WhitePaper_2006_SearchEngineUserBehavior.pdf. Accessed 27 Apr 2020

[20] Silberg WM, Lundberg GD, Musacchio RA (1997). Assessing, controlling, and assuring the quality of medical information on the Internet: caveant lector et viewor—let the reader and viewer beware. JAMA.

[21] Charnock D (1999). DISCERN: an instrument for judging the quality of written consumer health information on treatment choices. J Epidemiol Community Health.

[22] Diercks R (2014). Guideline for diagnosis and treatment of subacromial pain syndrome: a multidisciplinary review by the Dutch Orthopaedic Association. Acta Orthop.

[23] Babatunde OO (2021). Comparative effectiveness of treatment options for subacromial shoulder conditions: a systematic review and network meta-analysis. Ther Adv Musculoskelet Dis.

[24] Shire AR (2017). Specific or general exercise strategy for subacromial impingement syndrome-does it matter? A systematic literature review and meta analysis. BMC Musculoskelet Disord.

[25] Alqunaee M, Galvin R, Fahey T (2012). Diagnostic accuracy of clinical tests for subacromial impingement syndrome: a systematic review and meta-analysis. Arch Phys Med Rehabil.

[26] Gebremariam L (2011). Effectiveness of surgical and postsurgical interventions for the subacromial impingement syndrome: a systematic review. Arch Phys Med Rehabil.

[27] Dorrestijn O (2009). Conservative or surgical treatment for subacromial impingement syndrome? A systematic review. J Shoulder Elbow Surg.

[28] MacLeod MG (2015). YouTube as an information source for femoroacetabular impingement: a systematic review of video content. Arthroscopy.

[29] Kunze KN (2019). Youtube as a source of information about the posterior cruciate ligament: a content-quality and reliability analysis. Arthrosc Sports Med Rehabil.

[30] KLINIK-am-RING-Köln (2021) Impingement-Syndrom – Schulter – Ausg. 13. https://klinik-am-ring.de/orthopaedie/im-focus/impingement-syndrom-schulter/. Accessed 23 May 2021

[31] Hawkins R (2010) Shoulder Impingement—Dr. Richard Hawkins. https://www.youtube.com/watch?v=vARsKXb7wNc. Accessed 23 May 2021

[32] Zheluk A, Maddock J (2020). Plausibility of using a checklist with youtube to facilitate the discovery of acute low back pain self-management content: exploratory study. JMIR Form Res.

[33] Aydin MF, Aydin MA (2020). Quality and reliability of information available on YouTube and Google pertaining gastroesophageal reflux disease. Int J Med Inform.

[34] Koller U (2016). YouTube provides irrelevant information for the diagnosis and treatment of hip arthritis. Int Orthop.

[35] Staunton PF (1976). Online curves: a quality analysis of scoliosis videos on youtube. Spine.

[36] Fernandez-Llatas C (2017). Are health videos from hospitals, health organizations, and active users available to health consumers? An analysis of diabetes health video ranking in youtube. Comput Math Methods Med.

[37] Drozd B, Couvillon E, Suarez A (2018). Medical youtube videos and methods of evaluation: literature review. JMIR Med Educ.

[38] Wong K (2017). Youtube videos on botulinum toxin A for wrinkles: a useful resource for patient education. Dermatol Surg.

[39] Gokcen HB, Gumussuyu G (2019). A quality analysis of disc herniation videos on youtube. World Neurosurg.

[40] Morahan-Martin JM (2004). How internet users find, evaluate, and use online health information: a cross-cultural review. Cyberpsychol Behav.

