# Supplementary material for: Patient education on subacromial impingement syndrome: Reliability and educational quality of content available on Google and YouTube
Source: Orthopadie (Heidelb). 2022 Aug 22;51(12):1003–9. doi: 10.1007/s00132-022-04294-x (PMC9715471; doi:10.1007/s00132-022-04294-x)
Supplement: Supplementary file 1 — Subacromial Impingement Syndrom Score Evaluation Sheet [file 132_2022_4294_MOESM1_ESM.pdf]

Category	Points
**Definition**	**5**
**Etiology / pathogenesis**	**20**
Narrowness of the subacromial space	5
Possible causes: 1) structural; 2) functional	2 each
Structural bony causes:<br>1. Bone spurs<br>2. Hooked acromial shape	1 each
Different physiological variations of acromial shapes are possible	2
Types of shape variants of the acromion:<br>1. Flat (type 1)<br>2. Curved (type 2)<br>3. Hook-shaped (type 3)	1 each
A differentiation between several types of impingement is possible (e.g. outlet / non-outlet impingement, eccentric / intrinsic impingement, posterosuperior / anterosuperior impingement, etc.)	2
Epidemiology:<br>- Athletes<br>- Occupational groups with overhead work	1 easch
Symptoms	15
Pain caused by movement	5
Radiating pain into the upper arm	2
Resting pain	2
Loss of movement	2
Accompanying pathologies:<br>- Bursitis<br>- Rotator cuff lesion<br>- Scapulothoracic dyskinesia<br>- Tendinitis of the long biceps tendon	1 each
**Diagnostics**	**19**
Clinical examination, ultrasound, x-ray and MRI	2 each
Clinical examination:<br>- Painful arc<br>- Neer test<br>- Hawkins test<br>- Other tests for the supraspinatus tendon	je 1
Ultrasound:<br>- Assessment of the soft tissue	2
X-Ray:<br>- Assessment of the bony structes<br>- Assessment of the acromiohumeral distance<br>- Tendinits calcarea	1 each
MRI:<br>Assessment of the soft tissue	2
**Differential diagnosis**<br>- Capsular pathologies<br>- Pathologies of the long biceps tendon<br>- Neurological pathologies<br>- Pathologies of the acromioclavicular joint<br>- Osteoarthritis	**10**<br>2 each
**Therapy**	**31**

Conservative therapy:<br>- Physiotherapy<br>- NSAIDs<br>- Local anasthesia<br>- Cortison<br>- Avoiding painful movement<br>- Reducing triggering movements<br>- Rest<br>- Kryotherapy<br>- Heat<br>- Electrotherapy<br>- Shock wave	1 each
Goals of physiotherapy:<br>- Muscle strengthening<br>- Widening of the joint space<br>- Improving posture<br>- Stretching	1 each
Indications for surgical therapy:<br>- If conservative therapy is unsuccessful<br>- In the case of relevant accompanying pathologies	1 each
Surgical therapy: arthroscopic subacromial decompression / acromioplasty according to Neer	5
Surgical procedure:<br>- Diagnostic arthroscopy<br>- Partial bursectomy of the subactomial space<br>- Resection of the anterior edge of the acromion or significant osteophytes<br>- Treatment of accompanying pathologies	1 each
Risks of the operation:<br>- Nerve lesions<br>- Vascular injury<br>- Stiffness<br>- infection	1 each
Postoperative Treatment:<br>- Pain-adapted postoperative immobilization of the shoulder<br>- Gradual active and passive mobilization through physiotherapy	1 each
**Total score**	**100**
